# LATE-NC aggravates GVD-mediated necroptosis in Alzheimer’s disease

**DOI:** 10.1186/s40478-022-01432-6

**Published:** 2022-09-03

**Authors:** Marta J. Koper, Sandra O. Tomé, Klara Gawor, Annelies Belet, Evelien Van Schoor, Jolien Schaeverbeke, Rik Vandenberghe, Mathieu Vandenbulcke, Estifanos Ghebremedhin, Markus Otto, Christine A. F. von Arnim, Sriram Balusu, Matthew B. Blaschko, Bart De Strooper, Dietmar Rudolf Thal

**Affiliations:** 1 Laboratory for Neuropathology, Department of Imaging and Pathology, Leuven Brain Institute (LBI), KU Leuven, Herestraat 49, 3000 Leuven, Belgium; 2 Laboratory for the Research of Neurodegenerative Diseases, Department of Neurosciences, Leuven Brain Institute (LBI), KU Leuven, Leuven, Belgium; 3 Center for Brain and Disease Research, VIB, Leuven, Belgium; 4 Laboratory for Neurobiology, Department of Neurosciences, Leuven Brain Institute (LBI), KU Leuven, Leuven, Belgium; 5 Laboratory for Cognitive Neurology, Department of Neurosciences, Leuven Brain Institute (LBI), KU Leuven, Leuven, Belgium; 6 Laboratory for Translational Neuropsychiatry, Department of Neuroscience, Leuven Brain Institute (LBI), KU Leuven, Leuven, Belgium; 7 Institute of Anatomy – Anatomy I, Johann Wolfgang Goethe University, Frankfurt am Main, Germany; 8 Department of Neurology, Ulm University, Ulm, Germany; 9 Department of Neurology, University of Halle, Halle, Germany; 10 Department of Geriatrics, Göttingen University, Göttingen, Germany; 11 Department of Electronics, Center for Processing Speech and Images, KU Leuven, Leuven, Belgium; 12 Department of Pathology, UZ Leuven, Leuven, Belgium; 13 Department of Geriatric Psychiatry, UZ Leuven, Leuven, Belgium

**Keywords:** Granulovacuolar degeneration, LATE-NC, TDP-43, Necroptosis, Cell death, pMLKL, pTau, Protein aggregation

## Abstract

It has become evident that Alzheimer’s Disease (AD) is not only linked to its hallmark lesions—amyloid plaques and neurofibrillary tangles (NFTs)—but also to other co-occurring pathologies. This may lead to synergistic effects of the respective cellular and molecular players, resulting in neuronal death. One of these co-pathologies is the accumulation of phosphorylated transactive-response DNA binding protein 43 (pTDP-43) as neuronal cytoplasmic inclusions, currently considered to represent limbic-predominant age-related TDP-43 encephalopathy neuropathological changes (LATE-NC), in up to 70% of symptomatic AD cases. Granulovacuolar degeneration (GVD) is another AD co-pathology, which also contains TDP-43 and other AD-related proteins. Recently, we found that all proteins required for necroptosis execution, a previously defined programmed form of neuronal cell death, are present in GVD, such as the phosphorylated necroptosis executioner mixed-lineage kinase domain-like protein (pMLKL). Accordingly, this protein is a reliable marker for GVD lesions, similar to other known GVD proteins. Importantly, it is not yet known whether the presence of LATE-NC in symptomatic AD cases is associated with necroptosis pathway activation, presumably contributing to neuron loss by cell death execution. In this study, we investigated the impact of LATE-NC on the severity of necroptosis-associated GVD lesions, phosphorylated tau (pTau) pathology and neuronal density. First, we used 230 human *post-mortem* cases, including 82 controls without AD neuropathological changes (non-ADNC), 81 non-demented cases with ADNC, i.e.: pathologically-defined preclinical AD (p-preAD) and 67 demented cases with ADNC. We found that Braak NFT stage and LATE-NC stage were good predictors for GVD expansion and neuronal loss in the hippocampal CA1 region. Further, we compared the impact of TDP-43 accumulation on hippocampal expression of pMLKL-positive GVD, pTau as well as on neuronal density in a subset of nine non-ADNC controls, ten symptomatic AD cases with (AD^TDP+^) and eight without LATE-NC (AD^TDP−^). Here, we observed increased levels of pMLKL-positive, GVD-exhibiting neurons in AD^TDP+^ cases, compared to AD^TDP−^ and controls, which was accompanied by augmented pTau pathology. Neuronal loss in the CA1 region was increased in AD^TDP+^ compared to AD^TDP−^ cases. These data suggest that co-morbid LATE-NC in AD impacts not only pTau pathology but also GVD-mediated necroptosis pathway activation, which results in an accelerated neuronal demise. This further highlights the cumulative and synergistic effects of comorbid pathologies leading to neuronal loss in AD. Accordingly, protection against necroptotic neuronal death appears to be a promising therapeutic option for AD and LATE.

**Supplementary Information:**

The online version contains supplementary material available at 10.1186/s40478-022-01432-6.

## Introduction

Alzheimer’s Disease (AD) is the most common form of dementia, accounting for up to 80% of dementia cases worldwide, and is characterized by progressive memory loss and impairment in other cognitive domains such as executive dysfunction [1]. AD comprises two major neuropathological hallmarks, i.e., senile plaques containing amyloid β-protein (Aβ) and neurofibrillary tangles (NFTs) consisting of abnormally phosphorylated tau protein (pTau) [39].

Accumulating evidence suggests that AD is usually accompanied by multiple co-pathologies, rather than constituting a strictly defined disease [3, 4, 60, 66, 67], that ultimately lead to neuronal loss. One of these co-pathologies is the neuronal accumulation of transactive response DNA-binding protein 43 (TDP-43), more recently considered to represent limbic-predominant age-related TDP-43 encephalopathy neuropathological changes (LATE-NC) [53]. LATE-NC is found in the limbic system of up to 70% of AD cases [46]. In disease, TDP-43 is phosphorylated and translocated to the cytoplasm, forming pathological aggregates [54, 83]. Importantly, the presence of LATE-NC in AD has been associated with smaller hippocampal volumes and worsened cognitive performance [35, 37]. Interestingly, patients with pTDP-43 inclusions were shown to have clinically more severe dementia, compared to those without TDP-43 pathology [35], highlighting LATE-NC, as a relevant comorbidity in AD. TDP-43 has also been consistently associated with severe neuronal loss, such as in hippocampal sclerosis [50], as well as in animal models that recapitulate AD and TDP-43 pathologies [22, 57]. However, few studies have assessed the association between neuronal cell death and the presence of TDP-43 proteinopathy in the AD brain [38, 42].

**Fig. 1:**
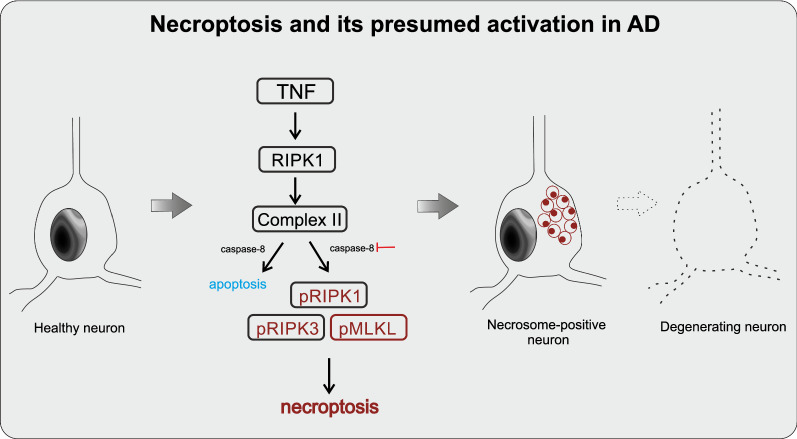
The necroptosis pathway and its presumed activation in AD. Necroptosis is a programmed form of cell death which starts with the activation of death receptors. RIPK1 is then recruited and self-phosphorylated [12]. The inhibition or activation of caspase-8 determines whether necroptosis or apoptosis takes place. If caspase-8 is inhibited, the necrosome complex is formed, which includes activated pRIPK1, pRIPK3 and pMLKL. This complex translocates to the cell membrane, resulting in membrane swelling and bursting mediated by pMLKL, the necroptosis executor [12]. This is followed by release of intracellular components, inflammatory response and ultimately neuronal death. Necroptosis activation has been previously linked to AD [12]. Here, the activated necrosome components pRIPK1, pRIPK3 and pMLKL accumulate in GVD granules presumably indicating an aberrant/delayed execution process of necroptosis in neurons with GVD [41]

Granulovacuolar degeneration (GVD) lesions are another comorbid pathology in AD [36], which are characterized by 3–5 µm vacuoles with dense granules of 0.5–1.5 μm dense core [21, 40, 65, 70]. Recently, we found that GVD granules also exhibit components of an activated programmed cell death pathway, specifically necroptosis, that correlated with neuronal loss in AD [41]. The activation of the canonical necroptosis pathway depends on the assembly of three proteins, including the phosphorylated receptor-interacting serine/threonine-protein kinase 1 and 3 (pRIPK1 and pRIPK3) [14] as well as the phosphorylated mixed lineage kinase domain-like protein (pMLKL) (Fig. 1) [13, 69]. The activation of necroptosis has been previously linked to AD [12]. Importantly, we found pMLKL to represent a reliable marker for GVD granules in AD, equal to pTDP-43 and CK1δ [41]. Once the activated proteins form the necrosome complex at the membrane, the cell swells, the membrane collapses, and cell death usually occurs within 24 h (Fig. 1) [62, 63]. Because the necrosome components are sequestrated in GVD granules [41], the execution of cell death is probably delayed, suggesting a specific form of necroptosis in AD-related neurodegeneration, which will be here referred to as GVD-mediated necroptosis.

Among the neurodegenerative processes in AD, pTau pathology is considered the most significant contributor to neuronal atrophy and cognitive decline [2, 52, 68]. Previously, we observed a strong correlation between pMLKL-positive GVD and Braak NFT stages [41]. Furthermore, we found that TDP-43 co-localizes and interacts with pTau lesions during AD progression [75], suggesting a synergy between these proteins [43]. In ALS/FTLD, we showed that TDP-43 and tau pathologies contributed to pMLKL-positive GVD in neurons of the CA1 subfield of the hippocampus [79]. However, it is not yet clear how local pTau and GVD severity changes upon the presence of LATE-NC in the AD brain. Thus, the question arises whether neuronal loss in AD cases presenting LATE-NC is associated with the necroptosis pathway, and whether the expression of activated necrosome markers and neuronal loss differs among AD cases with and without LATE-NC.

Here, we investigated *post-mortem* human brains of symptomatic AD cases with and without LATE-NC, hereby referred to as AD^TDP+^ and AD^TDP−^ cases, respectively, to clarify the impact of LATE-NC in necroptosis activation and neuronal death in AD. We also used a larger cohort including non-diseased individuals with and without AD neuropathological changes (ADNC) and demented cases with ADNC, with or without LATE-NC. We found that Braak NFT and LATE-NC stages were significantly associated with GVD stage, hippocampal neuronal loss, and dementia ratings. Moreover, we observed lower levels of neuronal death in symptomatic AD^TDP−^ cases, compared to AD^TDP+^. Interestingly, AD^TDP+^ cases exhibited exacerbated expression of pMLKL-positive GVD bodies when compared to AD^TDP−^ cases. Consistently, local pTau pathology was also aggravated in AD^TDP+^ cases. These data suggest that the presence of LATE-NC in AD accelerates neuronal cell death, specifically through a GVD-mediated form of necroptosis. TDP-43 probably affects pMLKL expression in GVD bodies-presumably indirectly-through its effect on pTau pathology. Our data highlight synergistic effects between TDP-43, pTau, and the necroptosis executor pMLKL. Thus, we emphasize the importance of LATE-NC co-pathology in the context of developing future therapeutic strategies targeting AD neurodegeneration.

## Material and methods

### Human samples

A total sample of 230 autopsy cases was investigated: 82 non-AD controls lacking AD neuropathological changes (ADNC) and signs of dementia, 81 non-demented individuals with ADNC considered to represent pathologically diagnosed preclinical AD (p-preAD) and 67 demented cases with ADNC. All autopsy brains were received from university or municipal hospitals in Leuven (Belgium), Bonn, Offenbach am Main and Ulm (Germany), and collected in accordance with local ethical committee guidelines and the federal laws governing the use of human tissue for research in Belgium and Germany. Dementia was diagnosed according to the DSM-IV criteria. The neuropathological diagnosis of ADNC was made when dementia was observed and when at least an intermediate degree of AD-related neuropathology was determined according to current criteria for the neuropathological diagnosis of ADNC as published by the National Institute of Aging and Alzheimer Association working group (NIA-AA criteria) [28]. The degree of dementia at the time of death was determined retrospectively using an estimate of the Clinical Dementia Rating (CDR) global score [26, 47]. For this, the information from the clinical files was used to provide a CDR global score. The CDR global score was applied in controls and AD cases with clinical symptoms [47]. Non-demented cases with ADNC, including cases with Aβ phase 1 or 2, were considered as pathologically-defined pre-clinical AD [58]. Primary-age related tauopathy (PART) was considered when cases presented AD-like pTau pathology in the absence of Aβ [15].

The left hemispheres were fixed in formalin for 2 to 4 weeks and dissected. Blocks from frontal, parietal, temporal, occipital, entorhinal cortex, the hippocampal formation at the level of the lateral geniculate body, basal ganglia, hypothalamus, thalamus, amygdala, basal nucleus of Meynert (NBM), midbrain, pons, medulla oblongata and cerebellum were embedded in paraffin. Five μm sections were cut using a microtome (Thermo Fisher Scientific).

### Selection criteria

Regarding the assessment of phases of Aβ deposition in the medial temporal lobe (AβMTL phases), Braak NFT, LATE-NC, GVD stages, CDR global score, neuronal density and APOE status, all available datasets from the entire cohort of 230 cases (Table 1) were used. Cases with clinically diagnosed ALS and cases younger than 18 years old were not included in the study.

**Table 1:** Study sample used for linear regressions, correlations and graphical lasso (N = 230)

Variable		Frequency	Mean
Age		–	67 years (range 25–96 years)
Male/Female		127 (55%)/ 103 (45%)	Not applicable
Neuropathological diagnosis	Non-ADNC	82 (37%)	Not applicable
	p-preAD	81 (36%)	
	Demented ADNC	67 (27%)	
Cognition	Non-impaired	83 (47%)	Not applicable
	Dementia	95 (53%)	
	Not available	52	
Braak NFT Stage	0	40 (17%)	II
	I	68 (30%)	
	II	36 (16%)	
	III	19 (8%)	
	IV	25 (11%)	
	V	20 (9%)	
	VI	22 (9%)	
AβMTL Phase	0	87 (38%)	2
	1	20 (9%)	
	2	20 (9%)	
	3	29 (12%)	
	4	74 (32%)	
LATE-NC Stage	0	132 (58%)	1
	1	10 (4%)	
	2	62 (27%)	
	3	26 (11%)	
GVD Stage	0	112 (51%)	1
	1	39 (18%)	
	2	12 (5%)	
	3	12 (5%)	
	4	28 (13%)	
	5	18 (8%)	
CDR Score	0	83 (47%)	1
	0.5	8 (4%)	
	1	22 (12%)	
	2	19 (11%)	
	3	46 (26%)	
	Not available	52	
CERAD Score	0	149 (65%)	1
	1	21 (9%)	
	2	34 (15%)	
	3	26 (11%)	
APOE status	2/2	1 (1%)	Not applicable
	2/3	14 (10%)	
	3/3	73 (53%)	
	3/4	40 (29%)	
	4/4	10 (7%)	
	Not available	91	

Number of cases assessed for each variable: age, sex, Braak NFT stage, AβMTL phase, LATE-NC stage, GVD stage, CERAD score = 230; Cognition/CDR score = 178; APOE status = 139

For the assessment of the impact of TDP-43 pathology on GVD-mediated necroptosis and neuronal loss in AD we selected 27 cases (Table 2) based on the presence/absence of LATE-NC in AD cases. Cases with the clinical diagnoses of frontotemporal dementia (FTD) with FTLD-TDP pathology, amyotrophic lateral sclerosis (ALS) cases or cases with other neurodegenerative diseases (FTLD-tau, Lewy Body dementia, Parkinson’s Disease) were excluded from the study. The number of cases was determined by availability of AD^TDP−^ cases. Similar numbers of non-AD controls and AD^TDP+^ cases were randomly chosen for each group. The nine controls were selected when Aβ pathology was absent and Braak stage was 0 or I. Therefore, the average age at death of this cohort (48 years) was lower when compared to that of the 230 cases (67 years). This is consistent with previous observations that cases lacking any ADNC are usually younger [9].

**Table 2:** Human autopsy cases used for quantification of neuronal density (CA1 hippocampus) and local accumulation of pMLKL, pTDP-43 and pTau

Case nr	Group	AβMTL Phase	Braak NFT Stage	Age	Sex	CDR Score	LATE-NC Stage	GVD Stage	Additional neuropathology	APOE status
1	Control	0	0	66	f	0	0	0	AS, AGD	3/3
2	Control	0	0	62	m	0	0	0	–	3/3
3	Control	0	1	46	m	0	0	0	–	3/3
4	Control	0	0	32	m	0	0	0	–	NA
5	Control	0	1	45	m	0	0	0	–	3/4
6	Control	0	0	18	m	0	0	0	–	3/3
7	Control	0	1	54	m	0	0	0	–	3/3
8	Control	0	1	59	f	3*	0	0	CAA	2/3
9	Control	0	0	67	m	0	0	0	–	3/3
10	AD^TDP−^	3	2	82	m	3	0	0	–	3/3
11	AD^TDP−^	3	4	79	f	NA	0	4	Microinfarcts	3/3
12	AD^TDP−^	4	4	87	f	3	0	3	AS, CAA	3/3
13	AD^TDP−^	3	3	85	m	2	0	2	CAA, microinfarcts	3/4
14	AD^TDP−^	3	3	81	m	2	0	1	AGD	3/3
15	AD^TDP−^	4	4	83	m	1	0	2	CAA, infarcts, microinfarcts	3/4
16	AD^TDP−^	4	4	72	f	1	0	2	Infarcts	3/3
17	AD^TDP−^	4	5	78	f	3	0	1	LBs, infarcts, SVD	4/4
18	AD^TDP+^	4	6	87	m	2	2	5	–	3/3
19	AD^TDP+^	4	5	71	m	3	2	3	–	4/4
20	AD^TDP+^	4	6	57	m	3	2	3	–	3/4
21	AD^TDP+^	4	5	76	m	3	3	5	ARTAG	3/4
22	AD^TDP+^	4	6	74	m	2	3	5	–	3/3
23	AD^TDP+^	4	5	81	f	3	2	5	CAA, infarcts	3/3
24	AD^TDP+^	4	5	78	f	3	2	5	–	3/4
25	AD^TDP+^	4	4	89	f	2	2	4	CAA, mild AS	3/4
26	AD^TDP+^	4	4	98	f	0.5	2	5	Infarcts, SVD	3/4
27	AD^TDP+^	4	5	89	f	3	2	4	CAA	3/4

^*^This case was rated with CDR score 3 because it presented lobar bleedings with consecutive epilepsy, albeit still a valid neuropathological control for AD

NA = not assessed; MTL = medial temporal lobe; NFT = neurofibrillary tangle; CDR = clinical dementia rating global score; LATE-NC = Limbic-predominant age-related TDP-43 encephalopathy neuropathological change; f = female; m = male; AS = atherosclerosis; AGD = argyrophilic grain disease; CAA = cerebral amyloid angiopathy; LB = Lewy body; SVD = small vessel disease; APOE = apolipoprotein E

### Neuropathology

For each case, Braak NFT stages, AβMTL phases, GVD and LATE-NC stages were determined by an experienced board-certified neuropathologist (DRT). AβMTL phases were determined as follows: Phase 0 is characterized by no detectable Aβ plaques. Aβ plaques in the temporal neocortex (layers III, V and VI) characterize AβMTL phase 1. In AβMTL phase 2, the plaque deposition spreads into the layers pre-β – pri-γ of the entorhinal cortex, CA1, and the subiculum. AβMTL phase 3 is characterized by Aβ deposition in all six layers of the temporal neocortex including subpial band-like Aβ accumulation. In addition, Aβ plaques also occur in the outer molecular layer of the dentate gyrus and the parvopyramidal cell layer of the presubicular region. Finally, in AβMTL phase 4 there is fully developed β-amyloidosis in the MTL with additional Aβ plaques in the CA4 region of the hippocampus and in the pre-α layer of the entorhinal cortex [73]. AβMTL phases correlate very well with the phases of Aβ deposition in the entire brain [72] and can be used as an alternative for the Aβ phases [28].

NFT distribution was assessed using the Braak NFT-staging method: stage 0 is characterized by absence of pTau NFTs, stage I by pTau-positive NFTs and threads, limited to the transentorhinal region, stage II by pTau pathology in the entorhinal region, extending to CA1 and CA2, stage III by affection of the neocortex of the fusiform and lingual gyri, stage IV by progression into neocortex-associated areas and the dentate gyrus, stage V by the involvement of frontal and occipital cortex, reaching the peristriate region (layer V also begins to be affected) and finally, stage VI is identified by pTau pathology in secondary and primary neocortical areas including the extension into the striate area of the occipital lobe [7, 8].

GVD stages were determined as previously described: stage 0 shows no GVD, stage 1 is characterized by GVD lesions restricted to the CA1/subiculum, in stage 2 GVD lesions progress to CA4 and the entorhinal cortex followed by temporal neocortex in stage 3. Stage 4 is characterized by affection of the amygdala and hypothalamus. Stage 5 is defined by the presence of GVD lesions in frontal and parietal neocortices [70].

The presence of LATE-NC was considered if one or more of the following lesions were identified: neuronal cytoplasmic inclusions, dystrophic neurites and neurofibrillary-tangle like material positive for antibodies against pTDP-43 (S409/S410) [32, 33]. Consistently, GVD was not considered as proper LATE-NC, and therefore if a case only presented GVD but no other pTDP-43 lesions, it was considered negative for LATE-NC. GVD lesions were quantified with an anti-pMLKL antibody.

Of note, the AD^TDP+^ cases used in the study all fit into Josephs’ TDP-43 pathological subtype β, which encompasses cases with TDP-43 lesions adjacent to/co-localizing with pTau NFTs [31].

LATE-NC stages were assessed according to recently published guidelines: the absence of LATE-NC corresponds to stage 0, in stage 1 TDP-43 lesions are present in the amygdala, followed by the hippocampal formation in stage 2 and expanding to the middle frontal gyrus in stage 3 [53].

APOE genotypes of these cases were obtained as described previously [71]. Briefly, DNA was extracted from fresh frozen or fixed paraffin-embedded tissue and PCR was performed followed by enzymatic digestion.

### Immunohistochemistry

The local expression and accumulation in the CA1 of pTDP-43 (S409/S410), pMLKL (S358) and pTau (S202/T205, clone AT8) were examined in 27 human samples of the hippocampus using immunohistochemical techniques (Table 2). Deparaffinization was performed in a robotized autostainer (Leica Microsystems) followed by antigen retrieval with EnVision Flex Target Retrieval Solution Low pH (citrate-based buffered solution at pH 6,1, Dako) in a PT Link Module (Dako). Blocking of endogenous peroxidase with EnVision FLEX Peroxidase-Blocking Reagent for 5 min was followed by a 30 min block with 5% bovine serum albumin (BSA). Tissue sections were incubated in a humid chamber overnight at room temperature (RT) with the primary antibodies. The information on all antibodies used in the study is summarized in Additional file 1: Table A1. Binding between primary antibody and corresponding HRP-conjugated secondary antibody (30 min, RT, Dako) was detected using 3,3'- diaminobenzidine solution (Liquid DAB + Substrate Chromogen System, Dako). Hematoxylin counterstaining and dehydration steps were carried out in the autostainer, followed by mounting in an automated cover-slipper (Leica Microsystems). Positive and negative controls were included in every staining procedure. The tissue sections were examined, and images were captured using a Leica DM2000 LED microscope equipped with a Leica DFC7000 T digital camera (Leica Microsystems).

### Immunofluorescence

Co-expression of the proteins of interest was studied by triple labeling immunofluorescence. Immunostainings were performed sequentially on hippocampal tissue using two antibodies raised in the same species. Primary antibodies (Additional file 1: Table A1) were detected with species-specific fluorescent-conjugated secondary antibodies (Jackson ImmunoResearch). The multiple rabbit-on-rabbit staining protocol was optimized based on previously described methods [24]. Briefly, a coupling method was used to avoid cross-reactivity of secondary antibodies when using primary antibodies from the same species. Tissue was incubated with a cocktail of anti-pTDP-43 and anti-pTau raised in rabbit and mouse, respectively, followed by fluorescent donkey anti-rabbit and anti-mouse secondary antibodies (Cy2 and Cy5, respectively). The second primary antibody raised in rabbit (pMLKL) was coupled to a donkey anti-rabbit Fab fragment (Jackson ImmunoResearch) conjugated to a fluorophore Cy3 (Jackson ImmunoResearch). For coupling, we incubated the primary antibodies with the respective Fab fragments for 20 min at RT (2 μg Fab fragment per 1 μg primary antibody). Next, normal rabbit serum (Jackson ImmunoResearch) was added to capture the unbound Fab fragments (10 μl of serum per 1 μg Fab fragment) for another 10 min. Thereafter, these conjugated primary antibodies were used to stain the second epitope. Hoechst 33342 staining was used for the visualization of nuclei (Thermo Fisher Scientific). Slides were manually mounted using Glycergel mounting medium (Dako). Image acquisition of hippocampal neurons was performed on Nikon NIS-Elements software using a Nikon A1R laser scanning confocal system attached to a Nikon Eclipse Ti inverted microscope (Nikon Instruments, Inc.).

### Quantification of neuronal density and protein accumulation

For a quantitative study, 27 cases were selected based on the presence/absence of TDP-43 pathology: Nine non-diseased controls without LATE-NC, eight neuropathologically-confirmed, symptomatic AD cases without LATE-NC (AD^TDP−^) and ten neuropathologically-confirmed, symptomatic AD cases with LATE-NC (AD^TDP+^) (Table 2). Immunoreactivity for pTDP-43 in GVD lesions was accepted in non-ADNC controls, AD^TDP−^ and AD^TDP+^ cases because GVD is not part of LATE-NC.

For this, three consecutive images (0.632 × 0.474 mm) of the CA1 subfield of the hippocampal formation immunoreactive for the specific antibody (pTDP-43, pMLKL or pTau) were captured with a 20 × objective lens on the Leica DM2000 LED microscope coupled to the DFC 7000 T camera. Neurons were identified based on the morphology and nuclear pattern of hematoxylin counterstaining. Neurons with morphological features indicating cell death or nuclear damage/absence were excluded from the analysis. An open-source image analysis software (Fiji/ImageJ) was used to quantify neuronal density (displayed in neurons/mm^2^) as well as ratios of pTDP-43-, pMLKL- and pTau-positive neurons relative to the total number of neurons (displayed in percentage). Full hippocampal overviews of two AD^TDP−^ and two AD^TDP+^ cases can be found in the public repository BioImage Archive, through the following hyperlink: https://www.ebi.ac.uk/biostudies/studies/S-BIAD514?key=475a3bbe-6fc9-476e-8e45-6429422b85bf.

### Statistical analysis

Statistical analysis was conducted in GraphPad Prism (version 9), SPSS (version 28) and R software (version 4.1.1). Neuronal density and the percentage of positive neurons for each antibody were compared across three groups using Kruskal–Wallis test. Nonparametric pairwise comparisons between groups were performed using the post-hoc Dunn’s test. Unpaired t-test was used to compare Braak NFT stages among AD^TDP−^ and AD^TDP+^ cases. Semi-partial correlation coefficients *(r)* based on Spearman’s rank correlations holding age and sex constant were calculated using ppcor R package. The results of significance tests were corrected for multiple comparisons using the Holm–Bonferroni method. Regression models were created to analyze the effect of explanatory variables (i.e., Braak NFT and LATE-NC stages) on each specific dependent variable. The results are presented as mean ± SEM. Statistical significance was preset at an α level of 0.05. To estimate the interaction strength between our parameters and remove spurious relations caused by indirect effects we computed a sparse Gaussian graphical model using graphical lasso [20]. This regularization was done on Spearman partial correlation matrix and the tuning parameter was chosen based on the extended Bayesian information criterium (EBIC) [19] using the EBICglasso function from qgraph R package. To ensure high specificity, we used the recommended value of the EBIC hyperparameter of 0.5 and elements of the inverse variance–covariance matrix had been thresholded following Jankova et al*.* [29]. We estimated the stability of the network by calculating correlation stability (CS) coefficient for nodes strength and edge weights with corStability from bootnet R package [18].

## Results

### Spreading of LATE-NC and pTau pathologies is associated with GVD expansion, neuronal loss, and cognitive status

To address whether the spread of LATE-NC and pTau pathologies (i.e., LATE-NC and Braak NFT stages) impact the severity of GVD, neuronal loss and dementia scores in AD, we used 230 human autopsy cases, covering 82 non-ADNC controls, 81 non-demented p-preAD cases with ADNC but without clinical signs of dementia and 67 demented cases with ADNC (Table 1). From these cases, 64 were also assessed for neuronal density in the CA1 subfield of the hippocampal formation and 178 were retrospectively assessed for clinical dementia rating (CDR), indicating the cognitive status of the individuals. The age distribution of this cohort is depicted in Additional file 1: Fig. A1a, showing that age ranges overlap among controls, p-preAD and AD cases.

**Fig. 2:**
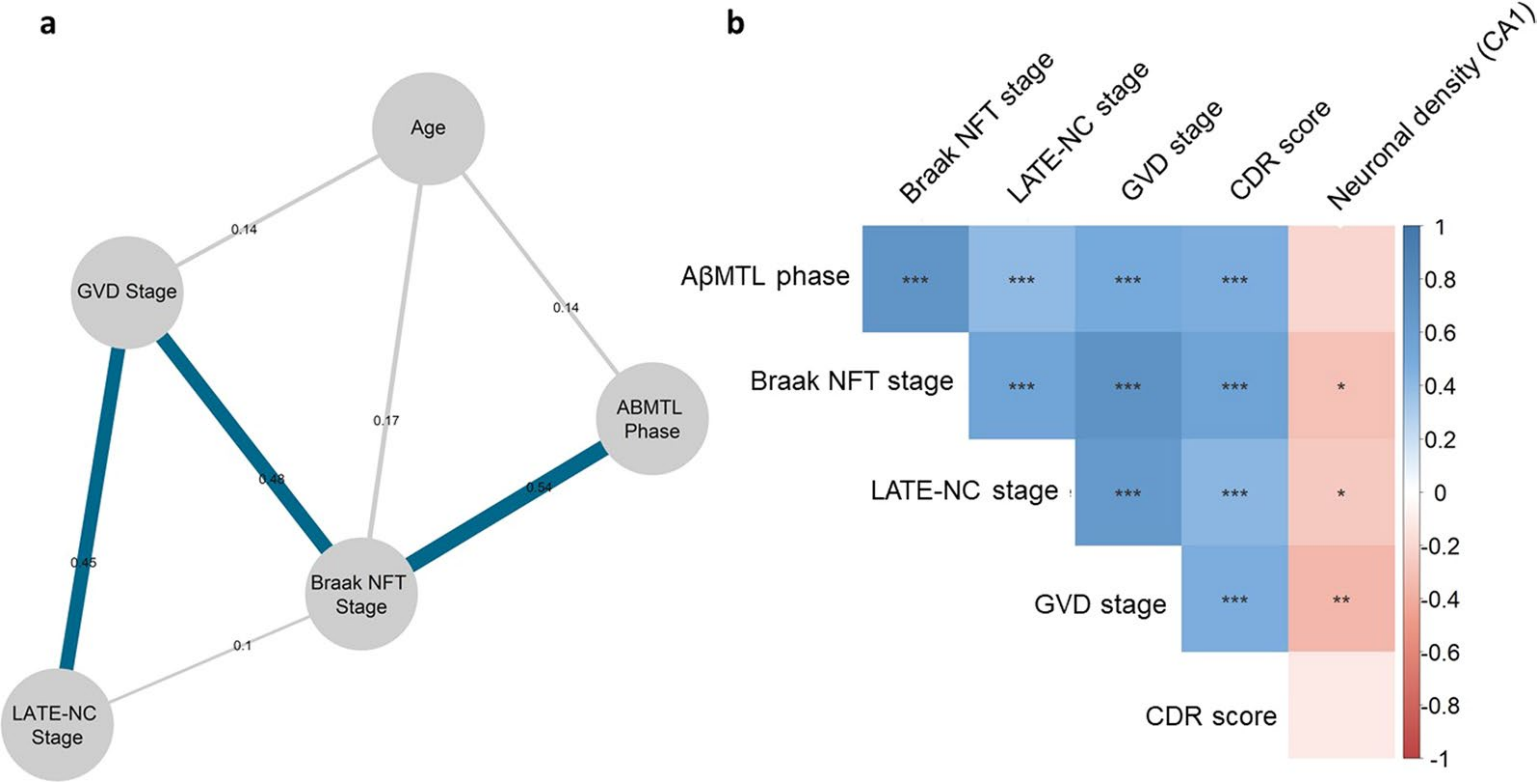
Tau, LATE-NC and GVD expansion are strongly associated. **a** Network constructed with graphical LASSO reveals that GVD is strongly associated with Braak NFT stage and mediates its relationship with LATE-NC stage (n = 221). The presence of connecting lines, i.e., edges between nodes (variables) indicates a positive and non-spurious partial correlation coefficient (with *r* values displayed on the edges and represented by the width of an edge). Edges representing correlation coefficients higher than 0.4 are marked in blue. **b** Semi-partial correlation matrix adjusted for sex shows that LATE-NC and Braak NFT stages are significantly correlated with GVD stage and CDR score, and that Braak NFT, LATE-NC and GVD stages negatively correlates with CA1-hippocampus neuronal density (n = 64). The *p*-values were adjusted with the Holm–Bonferroni method

First, we performed a network analysis with Spearman partial correlations and graphical lasso regularizations, including demented and non-demented individuals (Fig. 2a, n = 221). We identified seven edges (i.e., lines) indicating positive relations between nodes (i.e., variables). The strongest association was observed between AβMTL phase and Braak NFT stage (*r* = 0.54). Braak NFT stage was also strongly associated with GVD stage (*r* = 0.48), which in turn, exhibited a significant association with LATE-NC stage (*r* = 0.45). LATE-NC stage was also directly related to Braak NFT stage, however the effect was weaker (*r* = 0.1). Age was associated with both Braak NFT stage (*r* = 0.17), GVD stage (*r* = 0.14), and AβMTL phase (*r* = 0.14). (Fig. 2a). Stability analysis showed that both nodes strength and edge weights are sufficiently stable in this network (CS(cor = 0.7) > 0.5) and can support our conclusions.

Semi-partial correlations corrected for age and sex in all 230 cases corroborated these data and revealed that Braak NFT and LATE-NC stage were significantly correlated with GVD stage and CDR score (Fig. 2b). Braak NFT, LATE-NC and GVD stages also showed significant inverse correlations with neuronal density in the CA1-hippocampus (*r* = − 0.30, *r* = − 0.27, and *r* = − 0.36, n = 64). A negative correlation trend was observed between AβMTL phase, CDR and neuronal density, albeit not significant (Fig. 2b). Adjusted *p*-values and coefficient values are represented in Additional file 1: Tables A2a–b.

To validate these data, we performed logistic regression analyses controlled for age and sex. We found that LATE-NC and Braak NFT stages contribute independently to the expansion of GVD lesions when placed in the same model (*p* < 0.001*, β* = 0.313 and *β* = 0.596, *R*^*2*^ = 0.740, Additional file 1: Table A3). Moreover, we observed that LATE-NC and Braak NFT stages can predict neuronal density in the CA1 subfield of the hippocampus, albeit not independently from each other (*p* = 0.026, *β* = − 0.273, *R*^*2*^= 0.250 and *p* = 0.008, *β* = − 2.223*, R*^*2*^= 0.276; n = 64; Additional file 1: Tables A4a–c). When using LATE-NC stages and Braak NFT stages in a linear regression model to predict CDR score, only Braak NFT stage showed a significant association (*p* < 0.001, *β* = 0.562*,* *R*^*2*^ = 0.373*;* n = 178*;* Additional file 1: Table A5). LATE-NC stage also impacts CDR score, although not independently from Braak NFT stages (*p* < 0.001, *β* = 0.458, *R*^*2*^= 0.209; n = 178; Additional file 1: Tables A6a–b). Additionally, we observed that GVD expansion impacts neuronal density in CA1 (*p* = 0.006, *β* =  − 0.374, *R*^*2*^ = 0.290; n = 64; Additional file 1: Table A7). Of note, AβMTL phase did not have an independent effect on GVD expansion (*p* = 0.728, *β* = 0.020, *R*^*2*^ = 0.739; n = 230) or hippocampal neuronal density (*p* = 0.891, *β* = 0.032, *R*^*2*^ = 0.261; n = 64) when placed in a model with Braak NFT and LATE-NC stages (Additional file 1: Table A8 a–b).

### GVD-mediated necroptosis and pTau severity are increased in symptomatic AD cases with co-morbid LATE-NC

**Fig. 3:**
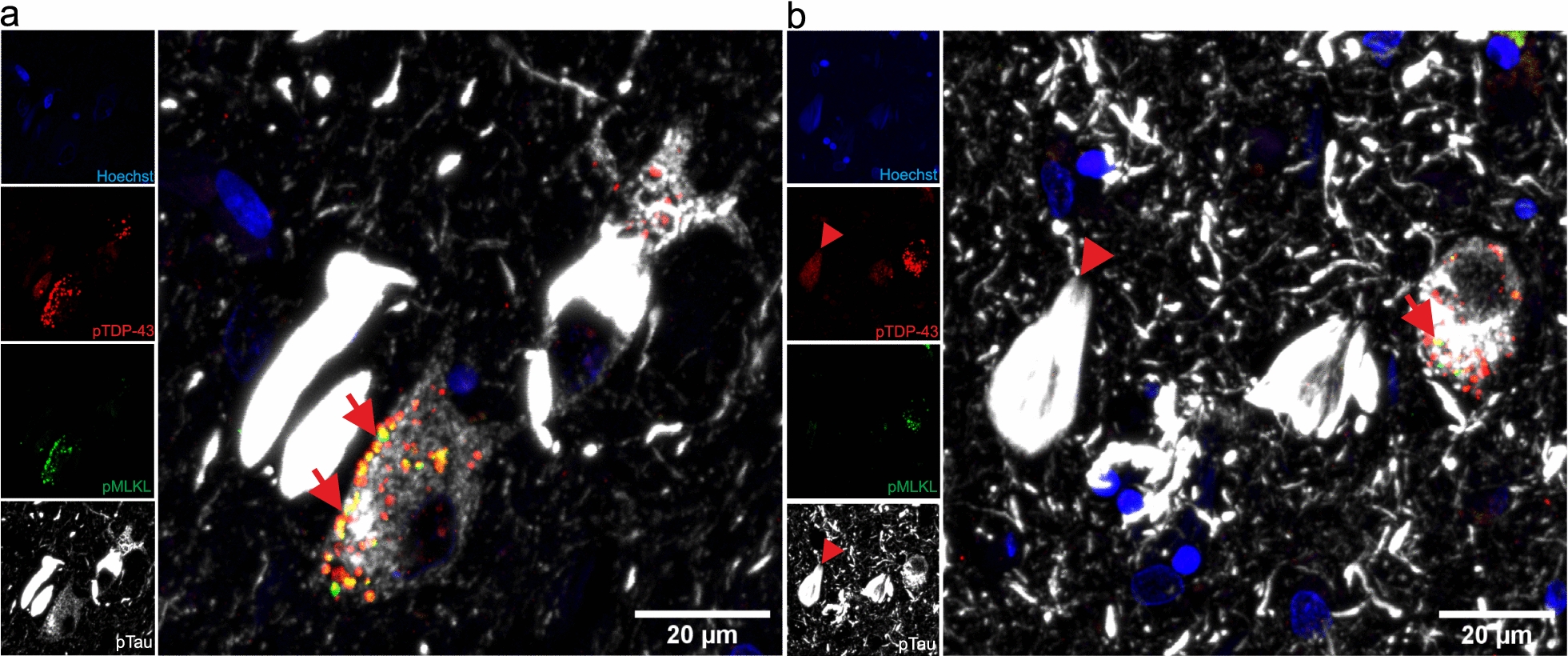
TDP-43 and pTau are co-expressed in necroptosis-positive neurons. Triple labeling immunofluorescence with antibodies against pTDP-43 (S409/S410), pTau (S202/T205) and pMLKL (S358) in the CA1 sub-hippocampal field of an AD^TDP+^ case. Nuclei are stained with Hoechst solution. Of note, **a** pTDP-43 and pMLKL are co-localized in some GVD granules (arrows) and **b** pTDP-43 and pTau co-localize in nearby neurons (arrowheads). We exclude the signal observed being considered as unspecific lipofuscin because such signal usually shows in the blue (Hoeschst) channel as orange granules, which in this case is absent

To address whether pTDP-43 and pTau are co-expressed in necrosome-positive GVD granules, we performed triple labeling in symptomatic AD cases with LATE-NC with anti-pTDP-43 (S409/S410), pTau (S202/T205, clone AT8) and pMLKL (S358), the effector protein of necroptosis. We observed that pMLKL and pTDP-43 proteins can co-localize within the same GVD granules, specifically in neurons bearing pTau pathology (Fig. 3a–b, arrows). Of note, pTau is also co-localizing with pTDP-43 in adjacent NFTs (Fig. 3b, arrowheads).

**Fig. 4:**
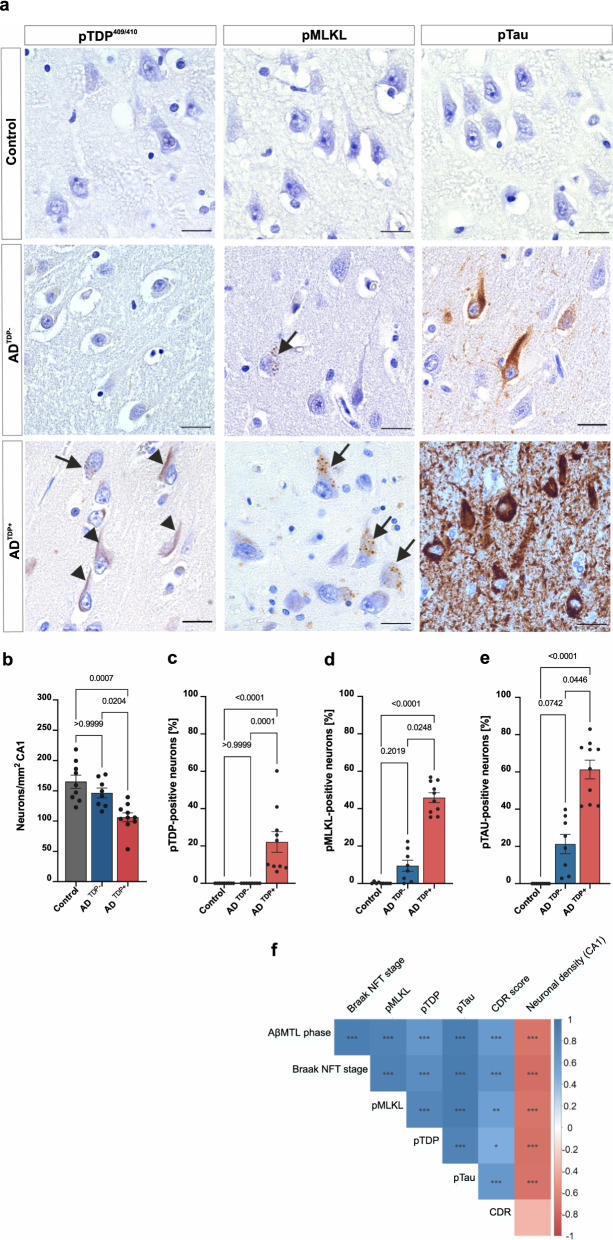
pMLKL and pTau severity as well as neuronal loss are increased in AD cases with LATE-NC. **a** Local accumulation of phosphorylated tau and MLKL is observed in AD^TDP+^. DAB immunohistochemical staining of pTDP-43 (S409/S410), pTau (S202/T205) and pMLKL (S358) in the CA1-subiculum field of a control, AD^TDP−^ and AD^TDP+^ case (cases 7, 16 and 21 are displayed, see Table 2). Scale bars = 50 µm. **b** AD^TDP+^ cases display decreased neuronal density compared to controls and AD^TDP−^ and controls. Quantitative data representing the number of total neurons per mm^2^ per group in the CA1 subfield of the hippocampus. Quantification of the number of CA1 positive neurons for **c** pTDP-43 **d** pMLKL and **e** pTau. AD^TDP+^ cases display significantly higher severity of pMLKL and pTau lesions. Data are presented as mean ± SEM. N = 27 (controls = 9, AD^TDP−^ = 8, AD^TDP+^  = 10). **f** Partial Spearman correlation matrix controlled for age and multiple comparisons (Holm-Bonferroni test) in this cohort confirms that the accumulation of pTDP-43, pMLKL and pTau pathologies are significantly correlated with neuronal density in the CA1, AβMTL phase and Braak NFT stage. Supporting images showing overviews of the whole hippocampus of cases 12, 16, 21 and 22 (Table 2) stained with pMLKL can be found in the public repository BioImage Archive, with the hyperlink: https://www.ebi.ac.uk/biostudies/studies/S-BIAD514?key=475a3bbe-6fc9-476e-8e45-6429422b85bf

Next, we aimed at investigating the impact of LATE-NC on pMLKL-positive GVD severity, neuronal death, and pTau pathology. Nine non-demented, non-ADNC controls, eight symptomatic AD cases without LATE-NC (AD^TDP−^) and ten symptomatic AD cases with LATE-NC (AD^TDP+^). The presence of LATE-NC was defined by the presence of NCIs, NFT-like material, DNs or NIIs as described previously [32, 53, 54, 77]. These cases were histopathologically evaluated with the following antibodies: pTDP-43 to assess TDP-43 pathology, pMLKL to assess GVD-positive neurons and pTau to assess pTau pathology (Fig. 4a). We quantitatively assessed the total population of neurons as well as the percentage of positive neurons for each of these markers in the CA1 subfield of the hippocampus. For this, a ratio of the number of positive neurons relative to the total number of CA1 neurons was estimated and displayed in percentage. Symptomatic AD^TDP+^ cases displayed an average of 22.15% of pTDP-43 positive neurons (Fig. 4a arrowheads, c). Compared to AD^TDP−^ and controls, AD^TDP+^ cases showed a decreased neuronal density in the CA1 (AD^TDP+^, 106.3 ± 7.3 neurons/mm^2^; AD^TDP−^, 146.4 ± 8.1 neurons/mm^2^; *p* = 0.0204) whereas AD^TDP−^ cases did not significantly differ from control brains (165.0 ± 10.9 neurons/mm^2^; *p* > 0.9999) (Fig. 4b). Since necroptosis was shown to play a role in AD neurodegeneration, we examined whether the observed differences in neuronal demise upon the presence of LATE-NC can be explained by the involvement of the necroptosis pathway. Symptomatic AD^TDP+^ cases exhibited significantly more pMLKL-positive neurons (45.87% ± 2.64), when compared to AD^TDP−^ cases (9.47% ± 2.98; *p* = 0.025) and non-diseased controls (0.21% ± 0.15; *p* < 0.0001) (Fig. 4a arrows, d). Consistently, symptomatic AD^TDP+^ cases also exhibited enhanced local accumulation of pTau (61.29% ± 5.04) compared to AD^TDP−^ (21.27% ± 5.16; *p* = 0.045) and controls (0.00; *p* < 0.0001) (Fig. 4a, e), highlighting the impact of co-morbid LATE-NC on pTau pathology and consequently on GVD-mediated necroptosis activation. Symptomatic AD^TDP+^ cases had higher Braak NFT stages, compared to AD^TDP−^ (*p* = 0.0016; Additional file 1: Fig. A2a), and importantly, these groups did not differ in age at death (*p* = 0.8435, Additional file 1: Fig. A2b), excluding age as a factor in the differences observed in neuronal density, pTau and pMLKL severity. Additionally, they did not differ in the presence/absence of APOE Ɛ4 allele (*p* = 0.1679; Additional file 1: Fig. A2c, Table 2).

Finally, we performed semi-partial correlation analysis controlled for sex but not for age (as age distributions among groups do not overlap, Additional file 1: A1b). We validated the significant relationship between the severity of LATE-NC, pTau and pMLKL (Fig. 4f). AβMTL phase and Braak NFT stages also correlated with the percentage of neurons positive for pTDP-43, pTau and pMLKL. Conversely, neuronal density was negatively correlated with all aforementioned parameters (Fig. 4f). Adjusted *p*-values and coefficient values are represented in Additional file 1: Tables A9a–b.

## Discussion

**Fig. 5:**
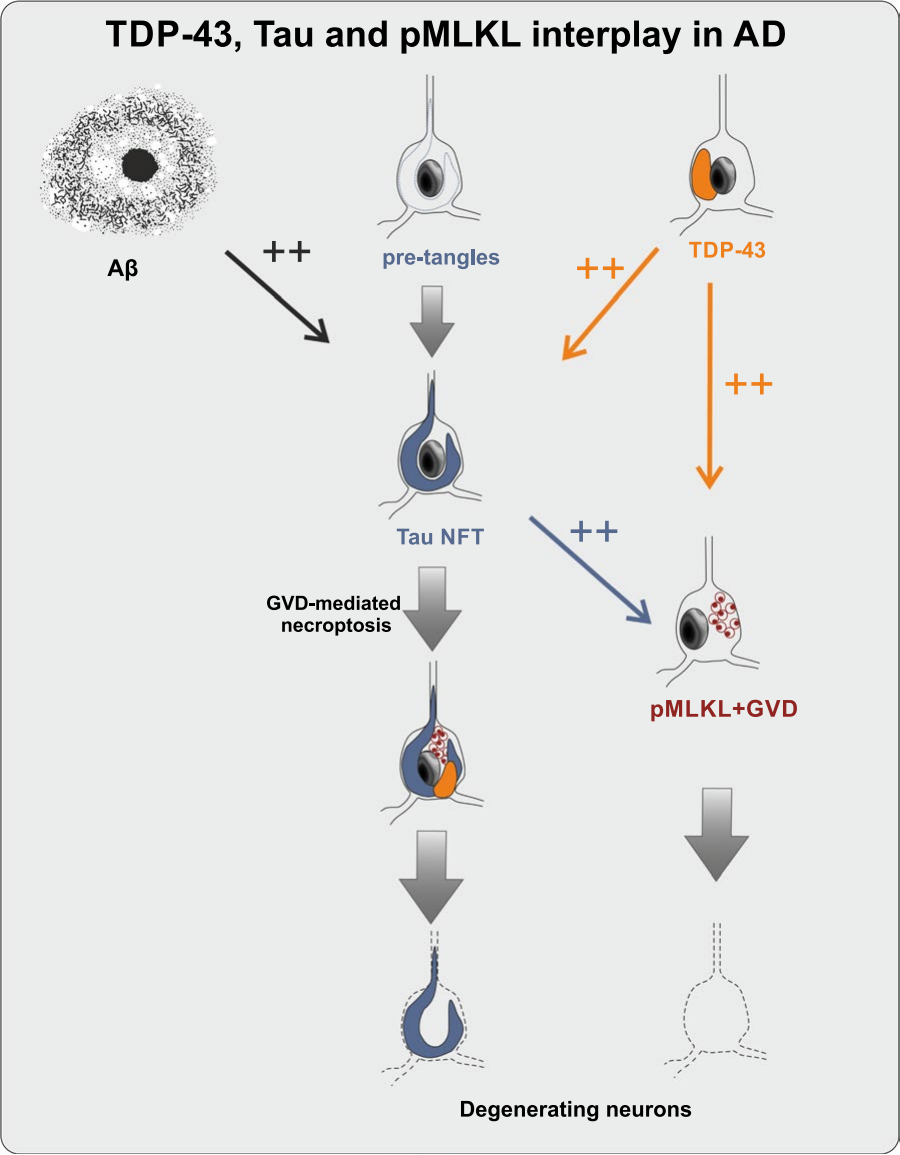
Proposed TDP-43, pTau and pMLKL interplay in AD. Aβ and TDP-43 pathologies accelerate pTau pathology in AD (black and orange arrows, respectively). We also hypothesize that TDP-43 exacerbates necrosome-positive GVD lesions through pTau (blue arrow), giving rise to neurons bearing tangles, pTDP-43 inclusions and pMLKL-positive GVD. This eventually leads to neurodegeneration and neuronal loss

In this study, we show that the presence of LATE-NC impacts neuronal loss in symptomatic AD cases. This is seemingly associated with the activation of the necroptotic pathway in GVD bodies, a form of cell death that we and others previously reported in AD [12, 41]. Accordingly, the presence of LATE-NC was associated with increased pTau and GVD burden, suggesting that LATE-NC may accelerate pTau pathology with subsequent induction of GVD-mediated necroptosis (Fig. 5).

Furthermore, we provide evidence that the spread of TDP-43 and pTau lesions is associated with the expansion of GVD bodies containing the activated necrosome, the clinical dementia rating global score and the reduction of hippocampal neuronal density. This is consistent with previous studies showing that LATE-NC actively contributes to the pathogenesis and clinical course of AD [35, 37, 49].

Importantly, we found higher expression of the necroptosis executor pMLKL in GVD bodies of symptomatic AD^TDP+^ compared to AD^TDP−^ cases, which was accompanied by enhanced local accumulation of pTau in the AD^TDP+^ group. Latimer and colleagues have recently shown that AD cases matched by Braak stage had increased pTau neocortical burden in the presence of comorbid TDP-43 proteinopathy [42], and also reported that TDP-43 promotes pTau aggregation in an animal model [44]. Additionally, Josephs et al*.* observed that AD cases with TDP-43 pathology have higher Braak NFT stages [35], which supports our results. Furthermore, we have previously provided evidence that pTau and TDP-43 interact during AD, probably facilitating the disease progression [75]. Other studies have also reported a strong interplay between these proteins in the pathogenic processes [16, 44]. Therefore, it seems that TDP-43 may exacerbate pTau pathology (Fig. 5, orange arrow) that is considered a crucial degenerative process promoting neuronal atrophy and cognitive decline in AD [52, 68]. This highlights TDP-43 as a relevant player in AD and is consistent with observations that co-morbid LATE-NC in AD worsens the clinical phenotype, compared to ADNC alone [35, 37, 51]. Aβ appears to play a smaller role in this context as it did not contribute separately to GVD expansion in addition to NFT and TDP-43 pathology although it is known to accelerate pTau propagation and aggregation [23, 25, 45].

The LATE-NC proteinopathy in AD is usually limbic-predominant, while the neocortex is frequently spared [33, 53]. Accordingly, once LATE-NC is present, pTau and TDP-43 pathologies increase in parallel in the medial temporal lobe [33]. Therefore, we speculate that TDP-43 accelerates pTau, possibly contributing to the development of “limbic-predominant subtype” and “typical AD” subtypes that were previously described, rather than the “hippocampal-sparing” subtype of AD [30, 34, 48].

The first report on the activation of necroptosis in AD revealed its positive association with Braak stage; whereas the correlation with cognitive function was negative [12]. Previously, we also analyzed the relationship between pMLKL-positive GVD and AD-defining parameters and observed the strongest association with Braak stage [41]. In line with this, we detected a higher number of pMLKL-positive GVD bodies in the hippocampus of symptomatic AD^TDP+^ cases, where pTDP-43, pTau and Aβ were present, compared to cases only exhibiting pTau and Aβ. Over the past decades, pTau was considered as a main inducer of GVD [5, 82], however recent reports highlight the role of other protein aggregates in this context such as TDP-43 [79] and *C9orf72* mutation-associated dipeptide repeat protein (DPR) in FTLD and ALS [59]. Our findings corroborate the hypothesis on the comorbid and accumulative effect of degenerative proteinopathies on the formation of GVD lesions, which in the context of AD intermediate the processing of necroptosis pathway activation and neuronal death.

The origin of GVD granules is not clear, however some authors describe them as pathologic cytoplasmic lesions resulting from a dysfunctional macroautophagic processing, and comprising deleterious post-translationally modified proteins [21, 40, 55]. It has been suggested that upstream intermingling processes induced by protein aggregates can initiate the development of GVD by impairing lysosomes that eventually fail to maintain cell homeostasis and become late-stage autophagic organelles under pathological conditions [21, 82]. pTDP-43 and pTau are known components of GVD granules [36, 82], which predominantly occur in ADNC but also to a lesser extent in non-AD tauopathies, including argyrophilic grain disease (AGD), progressive supranuclear palsy (PSP) and corticobasal degeneration (CBD), in TDP-43 proteinopathies, such as ALS and FTLD, as well as in normal aging [41, 70, 78, 79]. Here, TDP-43 pathology was accompanied by an exacerbated occurrence of pMLKL-positive GVD lesions and pTau pathology. This was associated with pronounced neuronal demise in the CA1 region of the hippocampus. When analyzing a larger cohort (n = 230 cases), we observed strong associations between NFT, GVD and LATE-NC stages. Moreover, hippocampal neuronal density was inversely correlated with Braak NFT as well as LATE-NC and GVD progression, supporting our results. Unexpectedly, age was not significantly associated with LATE-NC stages in this network. This was probably due to collinearity effects with Braak NFT, GVD, LATE-NC stages, and/or AβMTL phases, which were also included in this network and correlated with age.

Although we observed a positive association between the necroptosis pathway and LATE-NC in AD, there are reports corroborating this relationship in other diseases. Recently, we reported pMLKL-positive GVD granules to be correlated with proper TDP-43 pathology in the ALS/FTLD hippocampus [79]. Furthermore, there is evidence pointing that the absence of TDP-43 in oligodendrocytes triggers RIPK1-mediated necroptosis, which negatively affects the myelination process; having however no impact on motor neurons in an ALS/FTD model [80]. A recent study showed a protective function of TDP-43 that prevents an indirect activation of interferon-mediated necroptotic cell death [17]. In line with this, there was a report highlighting the mitochondrial impairment that is followed by TDP-43-associated cell death in the context of regulated necrosis [81]. Our findings, showing an enhanced GVD-mediated necroptosis in the presence of TDP-43 proteinopathy in symptomatic AD cases, point to the same role of TDP-43 pathological alterations leading to cell demise in different disease entities.

Therefore, this study underlines the urgency for developing reliable clinical biomarkers for TDP-43, which together with already available biomarkers for Aβ and Tau [6, 10, 56, 74] could help stratifying demented patients according to the presence and severity of underlying pathologies. GVD-mediated necroptosis associated with enhanced neuronal loss in AD patients with co-morbid LATE-NC seems to be exacerbated. We hypothesize that a cumulative effect of TDP-43 and Aβ in AD exacerbates pTau neurotoxicity, and thereby neuronal demise. Hence, detecting TDP-43 during life is of utmost importance to tailor treatments against neuronal death in AD patients.

A limitation of this study is the fact that this immunohistochemistry-based approach does not allow to conclude about functional, pathomechanistic pathways. However, the data obtained from the network analysis will help further functional studies to discover precise molecular mechanisms. Moreover, the number of symptomatic AD cases without LATE-NC was limited. The fact that this study was performed using hospital-based cohorts limits the interpretation regarding the epidemiology of these pathologies, as opposed to community-based cohorts. Moreover, hospital-based cohorts are usually enriched for disease and dementia as opposed to community-based studies [64], and individuals tend to present lower ages at death. Due to this, we also acknowledge the lack of relatively “pure” LATE-NC cases in our cohort, since most cases with LATE-NC had low to moderate levels of ADNC. However, the hospital-based sample approach still allows correlation, association, and network analysis of pathological parameters to determine their relationship with one another, as performed here. A final limitation is the low age at death of non-ADNC controls which is lower than in symptomatic cases with ADNC. The reason for this discrepancy in age at death is the result of the age-related frequencies of Aβ and pTau pathologies that explains why most cases without ADNC are 65 years of age or younger while symptomatic AD cases with end-stage ADNC are usually 70 years or older [9].

Taken together, our findings provide evidence for the synergy between pathologically aggregated proteins contributing to neuronal loss in AD, as already suggested by others [11, 27, 43, 61, 67]. Here, the absence of LATE-NC in patients with AD was associated with attenuated neuronal loss, even in the presence of intracellular pTau lesions and Aβ plaques. We hypothesize that TDP-43 contributes to the pathological cascade of AD by accelerating pTau pathology, probably by a direct interaction, which in turn induces cell death processes (i.e., GVD-mediated necroptosis) that contribute to the demise of neurons (Fig. 5). Therefore, our results argue in favor of developing personalized treatment strategies that consider co-morbid pathologies [43, 76], with GVD-mediated necroptosis being a presumably very important downstream target in this context.

## Supplementary Information

**Additional file 1.** **Table A1**–Antibodies used in this study. **Table A2**–P values (a) and coefficient values (b) of correlation matrix comparing AβMTL phase, Braak NFT stage, LATE-NC stage, GVD stage, CDR score and neuronal density in the CA1-hippocampus (n = 230). **Table A3**–LATE-NC and Braak NFT contribute independently to GVD expansion. Linear regression model including GVD stage as dependent variable and LATE-NC and Braak NFT stages, age and sex as independent variables is displayed (n = 230). **Table A4**–LATE-NC and Braak NFT contribute to CA1-hippocampus neuronal density, albeit dependently from each other (a-b). Linear regression models including neuronal density as dependent variable and LATE-NC and Braak NFT stages, age and sex as independent variables are displayed (n = 64). **Table A5**–Only Braak NFT stage contributes to dementia ratings. Linear regression model including CDR score as dependent variable and LATE-NC and Braak NFT stages, age and sex as independent variables is displayed (n = 178). **Table A6**–LATE-NC stage impacts CDR global score, but not independently from Braak NFT stage. Linear regression model using LATE-NC stage, age and sex as independent variables and CDR global score as dependent variable (n = 178). **Table A7**–GVD stage contributes significantly to neuronal density. Linear regression model including neuronal density as dependent variable and GVD stage, age and sex as independent variables is displayed (n = 64). **Table A8**–AβMTL phase does not contributes independently to (a) GVD stage or (b) neuronal density when placed in a model with Braak NFT and LATE-NC stages. Linear regression models including GVD stage or neuronal density in the CA1 as dependent variables and Braak NFT stage, LATE-NC stage, age and sex asindependent variables are displayed (a, n = 230; b, n = 64). **Table A9**–P values (a) and coefficient values (b) of correlation matrix comparing AβMTL phase, Braak NFT stage, CDR score, neuronal density in the CA1-hippocampus, as well as severity of pTDP, pMLKL and pTau severity. N = 26 (non-AD = 9, AD^TDP−^ = 8, AD^TDP+^ = 9). **Figure A1**–Age distribution in the cohorts used in this study a) n = 230, respective to Table 1 and b) n = 27, respective to Table 2. **Figure A2**–AD^TDP+^ cases have higher Braak NFT stage compared to AD^TDP−^ cases (a), but they do not differ inage at death (b) and (c) percentage of positive cases for an APOE Ꜫ4 allele. (a-b) Unpaired t-tests and means withSEM (n = 27); (c) Chi-square, two-sided test.

## Data Availability

The anonymized datasets used and/or analyzed during the current study are stored in UZ/KU-Leuven network drives and available from the corresponding author on reasonable request. Supporting images displaying overviews of the whole hippocampus stained with pMLKL of two AD^TDP+^ and two AD^TDP−^ cases are available in the public repository BioImage Archive through the following hyperlink: https://www.ebi.ac.uk/biostudies/studies/S-BIAD514?key=475a3bbe-6fc9-476e-8e45-6429422b85bf.
