# Supplementary material for: LATE-NC aggravates GVD-mediated necroptosis in Alzheimer’s disease
Source: Acta Neuropathol Commun. 2022 Sep 3;10:128. doi: 10.1186/s40478-022-01432-6 (PMC9441100; doi:10.1186/s40478-022-01432-6)

**Additional File 1 to:**

**LATE-NC aggravates GVD-mediated necroptosis in Alzheimer’s disease**

Marta J. Koper^1,2,3 *^, Sandra O. Tomé^1 *^, Klara Gawor^1^, Annelies Belet^1^, Evelien Van Schoor^1,3,4^, Jolien Schaeverbeke^1,5^, Rik Vandenberghe^5,6^, Mathieu Vandenbulcke^6,7^, Estifanos Ghebremedhin^8^, Markus Otto^9,10^, Christine A.F. von Arnim^9,11^, Sriram Balusu^2,3^, Matthew B. Blaschko^12^, Bart De Strooper^2,3^, Dietmar Rudolf Thal^1,13^.

^1^Laboratory for Neuropathology, Department of Imaging and Pathology, Leuven Brain Institute (LBI), KU Leuven, Leuven, Belgium

^2^Laboratory for the Research of Neurodegenerative Diseases, Department of Neurosciences, Leuven Brain Institute (LBI), KU Leuven, Leuven, Belgium

^3^Center for Brain and Disease Research, VIB, Leuven, Belgium

^4^Laboratory for Neurobiology, Department of Neurosciences, Leuven Brain Institute (LBI), KU Leuven, Leuven, Belgium

^5^Laboratory for Cognitive Neurology, Department of Neuroscience, Leuven Brain Institute (LBI), KU Leuven, Leuven, Belgium.

^6^Laboratory for Translational Neuropsychiatry, Department of Neuroscience, Leuven Brain Institute (LBI), KU Leuven, Leuven, Belgium

^7^Department of Geriatric Psychiatry, UZ Leuven, Leuven, Belgium

^8^Institute of Anatomy – Anatomy I, Johann Wolfgang Goethe University Frankfurt am Main, Germany

^9^Department of Neurology, Ulm University, Ulm, Germany

^10^Department of Neurology, University of Halle, Halle, Germany

^11^Department of Geriatrics, Göttingen University, Göttingen, Germany

^12^Department of Electronics – Center for Processing Speech and Images, KU Leuven, Leuven, Belgium

^13^Department of Pathology, UZ Leuven, Leuven, Belgium

*These authors contributed equally and are listed alphabetically

**Table A1 –** Antibodies used in this study.

| **Antibody name** | **Epitope** | **Source** | **Clonality; Clone no.** | **Dilution** | |
| --- | --- | --- | --- | --- | --- |
|  |  |  |  | **IHC** | **IF** |
| pTDP-43 | S409/pS410 | Cosmobio Co. LTD, Japan (TIP-PTD-P02) | Rabbit Polyclonal | 1:5000 | 1:1000 |
| pTau | S202/pT205 | ThermoFisher Scientific, US (MN1020) | Mouse Monoclonal; AT-8 | 1:1000 | 1:500 |
| pMLKL | S358 | Abcam, UK (ab187091) | Rabbit monoclonal | 1:100 | 1:50 |

**Table A2 –** *P* values **(a)** and coefficient values **(b)** of correlation matrix comparing AβMTL phase, Braak NFT stage, LATE-NC stage, GVD stage, CDR score and neuronal density in the CA1-hippocampus (n=230).

**a) *P*-values**

|  | Aβ Phase | Braak NFT Stage | LATE-NC Stage | GVD Stage | CDR Score | Neuronal Density |
| --- | --- | --- | --- | --- | --- | --- |
| AβMTL Phase | - |  |  |  |  |  |
| Braak NFT Stage | **p<0.0001** | - |  |  |  |  |
| LATE-NC Stage | **p<0.0001** | **p<0.0001** | - |  |  |  |
| GVD Stage | **p<0.0001** | **p<0.0001** | **p<0.0001** | - |  |  |
| CDR Score | **p<0.0001** | **p<0.0001** | **p<0.0001** | **p<0.0001** | - |  |
| Neuronal Density | 0.116242 | **0.017158** | **0.034089** | **0.006135** | 0.403666 | - |

**b) Coefficient values**

|  | Aβ Phase | Braak NFT Stage | LATE-NC Stage | GVD Stage | CDR Score | Neuronal Density |
| --- | --- | --- | --- | --- | --- | --- |
| AβMTL Phase | - |  |  |  |  |  |
| Braak NFT Stage | 0.707044 | - |  |  |  |  |
| LATE-NC Stage | 0.404771 | 0.550058 | - |  |  |  |
| GVD Stage | 0.502754 | 0.721421 | 0.657772 | - |  |  |
| CDR Score | 0.471952 | 0.573208 | 0.424098 | 0.478779 | - |  |
| Neuronal Density | -0.20153 | -0.30173 | -0.2696 | -0.35573 | -0.11711 | - |

**Table A3 -** LATE-NC and Braak NFT contribute independently to GVD expansion. Linear regression model including GVD stage as dependent variable and LATE-NC and Braak NFT stages, age and sex as independent variables is displayed (n=230).

| Model | Unstandardized Coefficients | | Standardized Coefficients |  |
| --- | --- | --- | --- | --- |
|  | B | Std. Error | β | Sig. |
| LATE-NC Stage | 0.49 | 0.074 | 0.313 | **<0.001** |
| Braak NFT Stage | 0.556 | 0.048 | 0.596 | **<0.001** |
| Age | 0.004 | 0.005 | 0.031 | 0.45 |
| Sex | 0.173 | 0.122 | 0.049 | 0.159 |
| **Dependent variable: GVD Stage** | | | | |
| **Adjusted R square = 0.740** | | | | |

**Table A4 -** LATE-NC and Braak NFT contribute to CA1-hippocampus neuronal density, albeit dependently from each other (a-b). Linear regression models including neuronal density as dependent variable and LATE-NC and Braak NFT stages, age and sex as independent variables are displayed (n = 64).

**a**

| Model | Unstandardized Coefficients | | Standardized Coefficients |  |
| --- | --- | --- | --- | --- |
|  | B | Std. Error | β | Sig. |
| LATE-NC Stage | -8.719 | 3.808 | -0.273 | **0.026** |
| Age | -0.921 | 0.335 | -0.335 | **0.008** |
| Sex | -7.677 | 8.079 | -0.107 | 0.346 |
| **Dependent variable: CA1 neuronal density** | | | | |
| **Adjusted R square = 0.250** | | | | |

**b**

| Model | Unstandardized Coefficients | | Standardized Coefficients |  |
| --- | --- | --- | --- | --- |
|  | B | Std. Error | β | Sig. |
| Braak NFT Stage | -6.095 | 2.102 | -2.223 | **0.008** |
| Age | -0.759 | 0.346 | -0.277 | 0.032 |
| Sex | -3.723 | 7.96 | -0.052 | 0.642 |
| **Dependent variable: CA1 neuronal density** | | | | |
| **Adjusted R square = 0.276** | | | | |

**c**

| Model | Unstandardized Coefficients | | Standardized Coefficients |  |
| --- | --- | --- | --- | --- |
|  | B | Std. Error | β | Sig. |
| LATE-NC Stage | -4.091 | 4.641 | -0.128 | 0.382 |
| Braak NFT Stage | -4.664 | 2.756 | -0.264 | 0.096 |
| Age | -0.725 | 0.349 | -0.264 | **0.042** |
| Sex | -5.022 | 8.109 | -0.07 | 0.538 |
| **Dependent variable: CA1 neuronal density** | | | | |
| **Adjusted R square = 0.273** | | | | |

**Table A5 -** Only Braak NFT stage contributes to dementia ratings. Linear regression model including CDR score as dependent variable and LATE-NC and Braak NFT stages, age and sex as independent variables is displayed (n=178).

| Model | Unstandardized Coefficients | | Standardized Coefficients |  |
| --- | --- | --- | --- | --- |
|  | B | Std. Error | β | Sig. |
| LATE-NC Stage | 0.142 | 0.087 | 0.13 | 0.104 |
| Braak NFT Stage | 0.37 | 0.054 | 0.562 | **<0.001** |
| Age | -0.009 | 0.007 | -0.085 | 0.204 |
| Sex | -0.139 | 0.155 | -0.054 | 0.37 |
| **Dependent variable: CDR score** | | | | |
| **Adjusted R square = 0.373** | | | | |

**Table A6 –** LATE-NC stage impacts CDR global score, but not independently from Braak NFT stage. Linear regression model using LATE-NC stage, age and sex as independent variables and CDR global score as dependent variable (n = 178).

**a) LATE-NC**

| Model | Unstandardized Coefficients | | Standardized Coefficients |  |
| --- | --- | --- | --- | --- |
|  | B | Std. Error | β | Sig. |
| LATE-NC Stage | 0.499 | 0.077 | 0.458 | **<0.001** |
| Age | 0.004 | 0.007 | 0.036 | 0.612 |
| Sex | -0.053 | 0.174 | -0.021 | 0.762 |
| **Dependent variable: CDR score**  **Adjusted R square = 0.209** | | | | |

**b) Braak NFT stage**

| Model | Unstandardized Coefficients | | Standardized Coefficients |  |
| --- | --- | --- | --- | --- |
|  | B | Std. Error | β | Sig. |
| Braak NFT Stage | 0.424 | 0.043 | 0.644 | **<0.001** |
| Age | -0.007 | 0.007 | -0.074 | 0.266 |
| Sex | -0.161 | 0.155 | -0.063 | 0.300 |
| **Dependent variable: CDR score**  **Adjusted R square = 0.367** | | | | |

**Table A7 -** GVD stage contributes significantly to neuronal density. Linear regression model including neuronal density as dependent variable and GVD stage, age and sex as independent variables is displayed (n=64).

| Model | Unstandardized Coefficients | | Standardized Coefficients |  |
| --- | --- | --- | --- | --- |
|  | B | Std. Error | β | Sig. |
| GVD Stage | -6.923 | 2.442 | -0.374 | **0.006** |
| Age | -0.718 | 0.348 | -0.273 | 0.044 |
| Sex | 0.239 | 7.984 | 0.003 | 0.976 |
| **Dependent variable: CA1 neuronal density** | | | | |
| **Adjusted R square = 0.290** | | | | |

**Table A8 –** AβMTL phase does not contributes independently to (a) GVD stage or (b) neuronal density when placed in a model with Braak NFT and LATE-NC stages. Linear regression models including GVD stage or neuronal density in the CA1 as dependent variables and Braak NFT stage, LATE-NC stage, age and sex as independent variables are displayed (a, n = 230; b, n = 64).

**a) GVD stage**

| Model | Unstandardized Coefficients | | Standardized Coefficients |  |
| --- | --- | --- | --- | --- |
|  | B | Std. Error | β | Sig. |
| Braak NFT Stage | 0.542 | 0.062 | 0.581 | **<0.001** |
| AβMTL Phase | 0.020 | 0.058 | 0.020 | 0.728 |
| LATE-NC Stage | 0.493 | 0.075 | 0.314 | **<0.001** |
| Age | 0.003 | 0.005 | 0.028 | 0.514 |
| Sex | 0.175 | 0.123 | 0.050 | 0.155 |
| **Dependent variable: GVD stage** | | | | |
| **Adjusted R square = 0.739** | | | | |

**b) CA1 neuronal density**

| Model | Unstandardized Coefficients | | Standardized Coefficients |  |
| --- | --- | --- | --- | --- |
|  | B | Std. Error | β | Sig. |
| Braak NFT Stage | -5.096 | 4.193 | -0.289 | 0.229 |
| AβMTL Phase | 0.656 | 4.768 | 0.032 | 0.891 |
| LATE-NC Stage | -4.103 | 4.681 | -0.128 | 0.384 |
| Age | -0.740 | 0.369 | -0.270 | 0.049 |
| Sex | -5.099 | 8.197 | -0.071 | 0.536 |
| **Dependent variable: CA1 neuronal density** | | | | |
| **Adjusted R square = 0.261** | | | | |

**Table A9 –** *P* values (a) and coefficient values (b) of correlation matrix comparing AβMTL phase, Braak NFT stage, CDR score, neuronal density in the CA1-hippocampus, as well as severity of pTDP, pMLKL and pTau severity. N = 26 (non-AD = 9, AD^TDP-^ = 8, AD^TDP+^ = 9).

**a) *P*-values**

|  | Aβ Phase | Braak NFT Stage | pMLKL | pTDP | pTau | CDR | Neuronal Density |
| --- | --- | --- | --- | --- | --- | --- | --- |
| AβMTL Phase | - |  |  |  |  |  |  |
| Braak NFT Stage | **p<0.0001** | - |  |  |  |  |  |
| pMLKL | **p<0.0001** | **p<0.0001** | - |  |  |  |  |
| pTDP | **p<0.0001** | **p<0.0001** | **p<0.0001** | - |  |  |  |
| pTau | **p<0.0001** | **p<0.0001** | **p<0.0001** | **p<0.0001** | - |  |  |
| CDR | **p<0.0001** | **p<0.0001** | **0.00239** | 0.0189 | **0.000427** | - |  |
| Neuronal Density | **p<0.0001** | **p<0.0001** | **p<0.0001** | **p<0.0001** | **p<0.0001** | 0.0820 | - |

**b) Coefficient values**

|  | Aβ Phase | Braak NFT Stage | pMLKL | pTDP | pTau | CDR | Neuronal Density |
| --- | --- | --- | --- | --- | --- | --- | --- |
| AβMTL Phase | - |  |  |  |  |  |  |
| Braak NFT Stage | 0,91990 | - |  |  |  |  |  |
| pMLKL | 0,86339 | 0,88492 | - |  |  |  |  |
| pTDP | 0,66554 | 0,76567 | 0,83725 | - |  |  |  |
| pTau | 0,91380 | 0,93125 | 0,93574 | 0,86071 | - |  |  |
| CDR | 0,63530 | 0,71577 | 0,58368 | 0,46849 | 0,65723 | - |  |
| Neuronal Density | -0,70623 | -0,74965 | -0,70624 | -0,72015 | -0,71552 | -0,35455 | - |

**Figure A1 –** Age distribution in the cohorts used in this study a) n=230, respective to Table 1 and b) n=27, respective to Table 2.

**a**
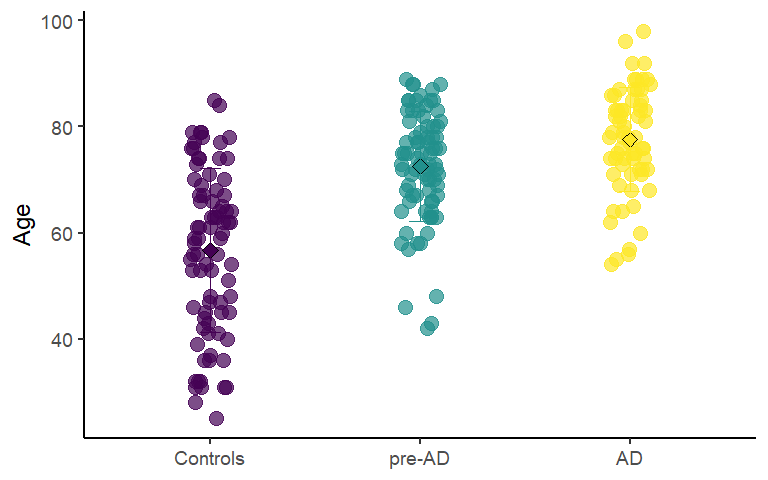


**b**


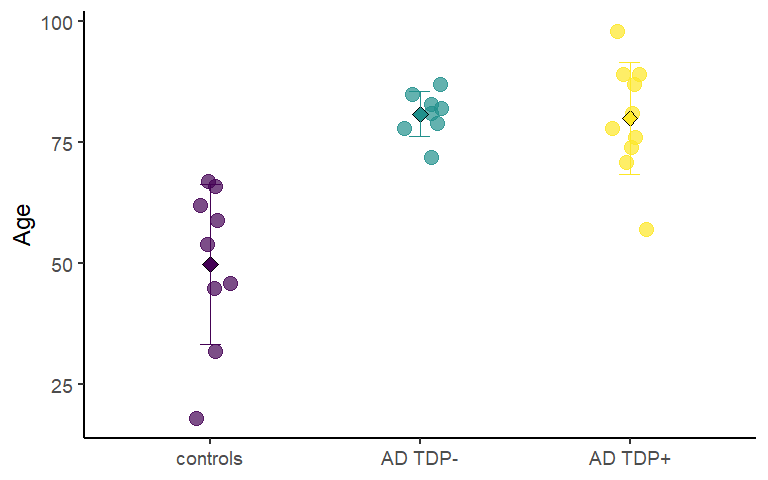

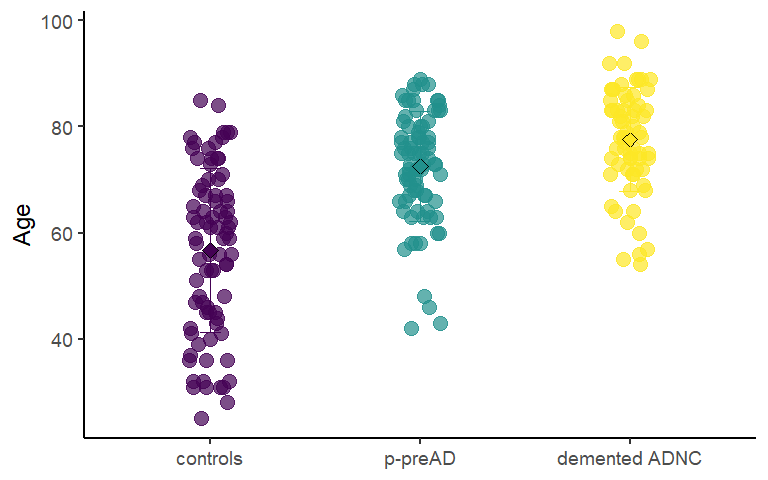


**Figure A2** –AD^TDP+^ cases have higher Braak NFT stage compared to AD^TDP-^ cases (a), but they do not differ in age at death (b) and (c) percentage of positive cases for an APOE Ꜫ4 allele. (a-b) Unpaired t-tests and means with SEM (n=27); (c) Chi-square, two-sided test.

**c**
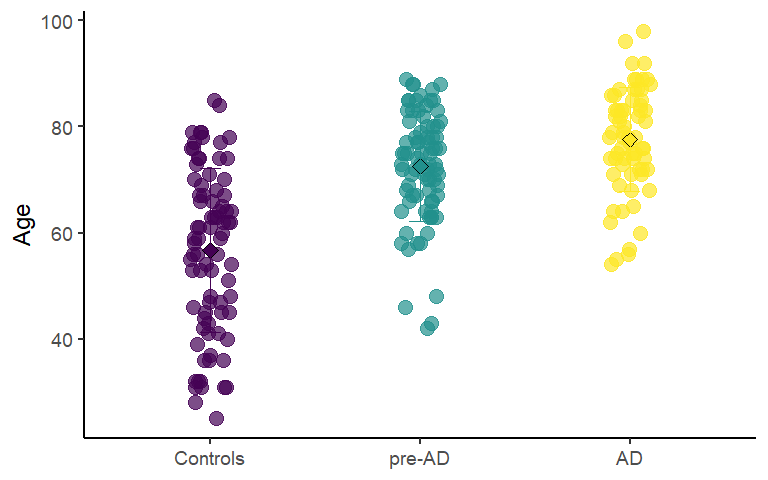


**b**
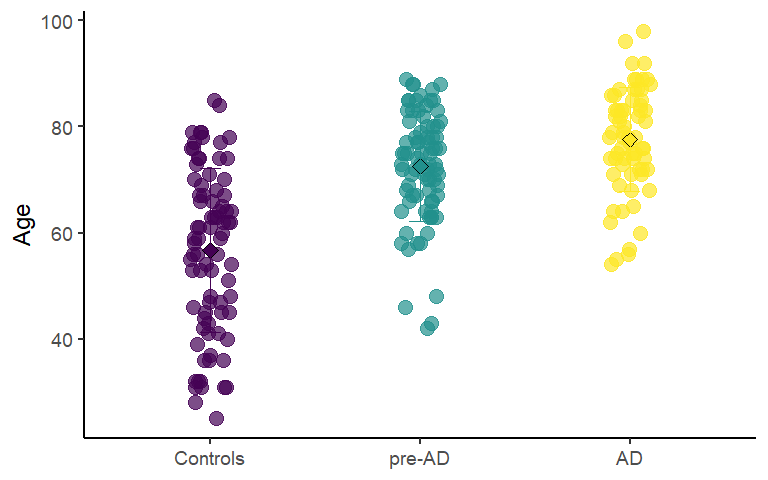


**a**
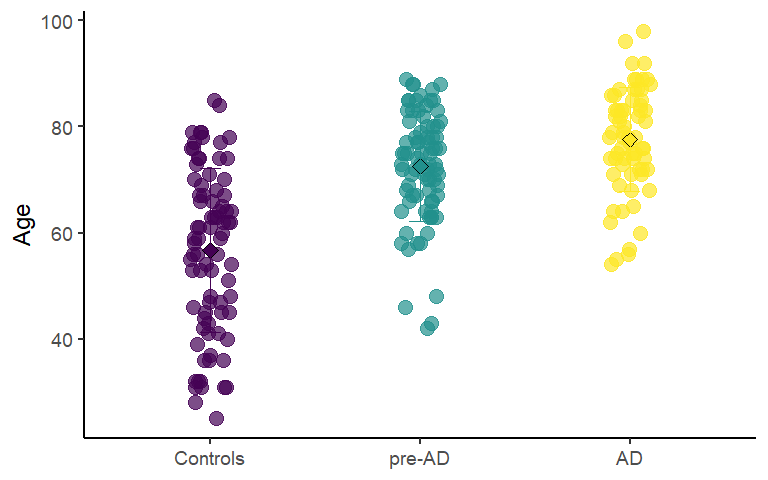

Supplement: Supplementary file 1 — Additional file 1. Table A1–Antibodies used in this study. Table A2–P values (a) and coefficient values (b) of correlation matrix comparing AβMTL phase, Braak NFT stage, LATE-NC stage, GVD stage, CDR score and neuronal density in the CA1-hippocampus (n = 230). Table A3–LATE-NC and Braak NFT contribute independently to GVD expansion. Linear regression model including GVD stage as dependent variable and LATE-NC and Braak NFT stages, age and sex as independent variables is displayed (n = 230). Table A4–LATE-NC and Braak NFT contribute to CA1-hippocampus neuronal density, albeit dependently from each other (a-b). Linear regression models including neuronal density as dependent variable and LATE-NC and Braak NFT stages, age and sex as independent variables are displayed (n = 64). Table A5–Only Braak NFT stage contributes to dementia ratings. Linear regression model including CDR score as dependent variable and LATE-NC and Braak NFT stages, age and sex as independent variables is displayed (n = 178). Table A6–LATE-NC stage impacts CDR global score, but not independently from Braak NFT stage. Linear regression model using LATE-NC stage, age and sex as independent variables and CDR global score as dependent variable (n = 178). Table A7–GVD stage contributes significantly to neuronal density. Linear regression model including neuronal density as dependent variable and GVD stage, age and sex as independent variables is displayed (n = 64). Table A8–AβMTL phase does not contributes independently to (a) GVD stage or (b) neuronal density when placed in a model with Braak NFT and LATE-NC stages. Linear regression models including GVD stage or neuronal density in the CA1 as dependent variables and Braak NFT stage, LATE-NC stage, age and sex asindependent variables are displayed (a, n = 230; b, n = 64). Table A9–P values (a) and coefficient values (b) of correlation matrix comparing AβMTL phase, Braak NFT stage, CDR score, neuronal density in the CA1-hippocampus, as well as severity [file 40478_2022_1432_MOESM1_ESM.docx]
